# The Prognostic Signature and Therapeutic Value of Phagocytic Regulatory Factors in Prostate Adenocarcinoma (PRAD)

**DOI:** 10.3389/fgene.2022.877278

**Published:** 2022-05-30

**Authors:** Shiyong Xin, Xianchao Sun, Liang Jin, Weiyi Li, Xiang Liu, Liqing Zhou, Lin Ye

**Affiliations:** ^1^ Department of Urology, Shanghai East Hospital, School of Medicine, Tongji University, Shanghai, China; ^2^ Department of Urology, Shanghai Tenth People’s Hospital, School of Medicine, Tongji University, Shanghai, China; ^3^ Department of Rheumatology and Immunology, The First Affiliated Hospital, and College of Clinical Medicine of Henan University of Science and Technology, Luoyang, China

**Keywords:** prostate cancer, PRAD, phagocytic factor, immune infiltration, survival analysis

## Abstract

There is growing evidence that phagocytosis regulatory factors (PRFs) play important roles in tumor progression, and therefore, identifying and characterizing these factors is crucial for understanding the mechanisms of cellular phagocytosis in tumorigenesis. Our research aimed to comprehensively characterize PRFs in prostate adenocarcinoma (PRAD) and to screen and determine important PRFs in PRAD which may help to inform tumor prognostic and therapeutic signatures based on these key PRFs. Here, we first systematically described the expression of PRFs in PRAD and evaluated their expression patterns and their prognostic value. We then analyzed prognostic phagocytic factors by Cox and Lasso analysis and constructed a phagocytic factor-mediated risk score. We then divided the samples into two groups with significant differences in overall survival (OS) based on the risk score. Then, we performed correlation analysis between the risk score and clinical features, immune infiltration levels, immune characteristics, immune checkpoint expression, IC50 of several classical sensitive drugs, and immunotherapy efficacy. Finally, the Human Protein Atlas (HPA) database was used to determine the protein expression of 18 PRF characteristic genes. The aforementioned results confirmed that multilayer alterations of PRFs were associated with the prognosis of patients with PRAD and the degree of macrophage infiltration. These findings may provide us with potential new therapies for PRAD.

## Introduction

As the second most prevalent malignancy in men worldwide, prostate cancer (PC) ranks as the fifth leading cause of male cancer deaths. According to the reports of the International Agency for Research on Cancer (IARC), there were estimated to be 1.276 million new cases of PC and approximately 359,000 deaths from PC worldwide in 2018. The age-standardized incidence rate by world standard population (ASIRW) and age-standardized mortality rate by world standard population (ASMRW) were 29.3 per 100,000 and 7.6 per 100,000, respectively (Bray et al., 2018). Treatment options for early-stage PC can be divided into non-radical treatments such as watchful waiting and hormone therapy and radical treatments such as radical prostatectomy, radiation therapy, and cryotherapy. The current first-line treatment option for patients with advanced metastatic PC still remains endocrine therapy, but after a median duration of 18–24 months of endocrine therapy, almost all patients will progress to castration-resistant PC (CRPC). Once patients enter this stage, the prognosis is poor. Due to the heterogeneous nature of PC, it remains difficult for urologists to develop individualized clinical treatment plans for their patients (Knight, 2014). Although drugs such as docetaxel, abiraterone, and apatamide have been developed for the treatment of CRPC, which can extend life expectancy to some extent, the low survival rate of mCRPC suggests it remains urgent for us to carry out further research into the mechanisms related to the development of PC, especially CRPC, and to develop novel targeted therapies.

Phagocytosis regulatory factors, as with common cytokines, are expressed in almost all cancers and promote six characteristics of cancer cells, thus promoting the occurrence of tumors. The tumor microenvironment provides the necessary immunosuppressive and hypermetabolic environment for tumor growth and progression. Tumor-associated macrophages (TAMs) play an important role in inhibiting tumor immunity, while PRFs play an important role in regulating the activity of induced tumor immune cells such as macrophages and T cells. Current studies have shown, in many cancers (liver, gastric, esophageal, colon, and bladder), that immune-infiltrating cells in a tumor microenvironment can promote tumor growth by promoting angiogenesis, cause DNA damage, and alter the microenvironment thus evading immune responses. Inflammatory cells can not only directly promote tumorigenesis, growth, and metastasis by remodeling the extracellular matrix (ECM) and mediating epithelial–mesenchymal trans differentiation (EMT) but also release cytokines further causing the promotion of tumor growth (Khan et al., 2016). Macrophages are innate immune cells and represent the main cell type of tumor-infiltrating immune cells. TAMs are a source of several inflammatory cytokines, and these cells can be divided into two types: tumor-suppressive M1 and tumorigenic M2 (Lanciotti et al., 2014). Current studies have focused on M2 cells, which contribute to tissue repair and vascular regeneration. Phagocytosis plays a critical role in the neutralization and termination of pathogens (Lim et al., 2017) but can also contribute to a diverse range of developmental, homeostatic, and non-infectious disease processes, including apoptotic cell clearance, senescent erythrocyte turnover, tumor surveillance, elimination of cellular debris after injury, and synaptic pruning (Arandjelovic and Ravichandran, 2015; Gordon, 2016; Hong et al., 2016; Chen et al., 2017). Dysregulation of phagocytosis by professional or non-professional phagocytes (Chung et al., 2013; Juncadella et al., 2013) can lead to autoimmunity, developmental deficits, and buildup of toxic protein aggregates (Gordon, 2016; Hong et al., 2016). Much of our current understanding of the molecular basis of phagocytosis is derived from forward genetic screens in model organisms. Numerous PRFs in cultured *Drosophila* S2 cells have been identified by RNAi screens (Kocks et al., 2005; Philips et al., 2005), although a systematic screen for PRFs in mammalian cells has not been reported.

Monoclonal antibody therapies targeting tumor antigens have been described as “magic bullets” for cancer therapy, which drive cancer cell elimination in part by triggering a macrophage-derived phagocytosis of cancer cells (Scott et al., 2012; Maleki et al., 2013; Sliwkowski and Mellman, 2013; Weiskopf and Weissman, 2015; Tsao et al., 2019). However, a low phagocytic activity of macrophages in the tumor microenvironment and the expression of anti-phagocytic factors by cancer cells, remain key obstacles before the promise of a monoclonal antibody therapy for diverse cancers can be realized (Ruffell and Coussens, 2015; Cunha et al., 2018; Su et al., 2018; Pathria et al., 2019). Furthermore, the mechanisms by which cancer cells evade phagocytosis are not fully understood. In a study published in Nature, researchers used intercellular CRISPR screening to identify PRFs in cancer cells, and using genome-wide reverse screening in macrophages, they found that the G-protein-coupled receptor GPR84 mediates enhanced phagocytosis in APMAP-deficient cancer cells. This work revealed an intrinsic cancer regulator that was sensitive to antibody-driven phagocytosis, expanding our understanding of the mechanisms by which cancers are resistant to the phagocytosis of macrophages (Roarke et al., 2021).

In PC, numerous controlled clinical trials and meta-analyses have confirmed that inflammatory cells and related factors can promote the growth of PC cells (Mishina et al., 1985). Lanciotti et al. (2014) found that in pathological sections from PC patients treated with androgen deprivation therapy (ADT), the group with lower TAM counts (<22) had a significantly better prognosis than the group with higher counts (>22) (Nonomura et al., 2011), suggesting that TAMs may be a good predictive marker for PC. In addition, if the replenishment of macrophages in the prostate is blocked, the incidence of PC can be reduced to some extent and the development of mCRPC can be delayed, suggesting that the use of NSAIDs can reduce the risk of PC (Mantovani et al., 2006). However, the specific role of other subtypes of macrophages and associated PRFs are yet to be resolved, except for M2, which requires further study. As with macrophages, cytokines such as PRFs may play an important role in the occurrence and development of tumors. However, thus far, the mechanistic role of PRFs in PC has not been reported. Therefore, further studies on the relevance of immune-infiltrating inflammatory cells, especially macrophages and related PRFs, and the tumor microenvironment are of obvious importance for the further treatment of PC.

**FIGURE 1:**
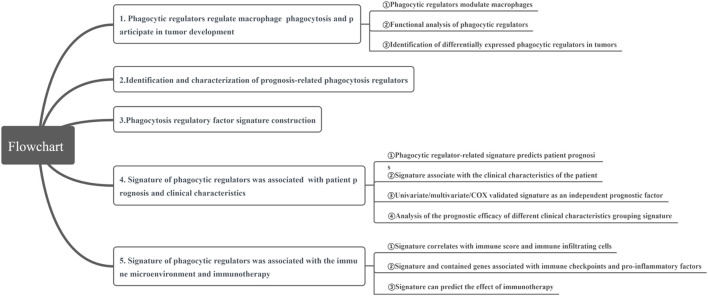
The flowchart of this study.

In this study, we aimed to comprehensively characterize PRFs in PRAD, and to screen and determine key PRFs, and construct tumor prognostic and therapeutic signatures based on the relevant genes. First, we systematically described the expression of PRFs in prostate PRAD, and evaluated their expression patterns and their relationship with patient outcome. We then analyzed potential prognostic PRFs by using the Cox and Lasso analysis and constructed a phagocytic factor-mediated risk core. We then divided the samples into two groups with significant differences in OS prognosis based on the risk core. Finally, a correlation analysis was conducted between the risk score and clinical features, immune infiltration molecules, immune characteristics, immune checkpoint expression, IC50 of several classical sensitive drugs, and immunotherapy efficacy. Meanwhile, the Human Protein Atlas (HPA) database was used to determine the protein expression of 18 PRF characteristic genes. Through our study, we aimed to clarify the role of PRFs in the development of PRAD, especially in castration resistance, and to explore the role of PRFs in the tumor microenvironment of PRAD, and to discuss their value in treatment and prognosis prediction (Figure 1).

## Data and Methods

### Study Data

Based on the published literature, 173 PRFs were collected, of which 171 phagocytosis regulatory genes involved in PRAD had expression information submitted to The Cancer Genome Atlas (TCGA) (Haney et al., 2018; Roarke et al., 2021). TCGA data such as the mRNA expression profile data of phagocytosis regulatory factors and survival information for PRAD were downloaded from https://xenabrowser.net/datapages, and clinical information was downloaded using the R package cgdsr, and 532 of these RNA-seq samples with survival data were screened. The summary of the survival information for these patients is shown in Supplementary Table S1. In addition, we downloaded one set of PRAD samples with the expression profile data and their generation information from GEO (https://www.ncbi.nlm.nih.gov/geo/) for model validation. Furthermore, immunotherapy data were used from the Bladder Cancer (BLCA) dataset (IMvigor210CoreBiologies) and the Kidney Clear Cell Carcinoma (KIRC) dataset (PMID32472114), and information on the BLCA samples is shown in Supplementary Table S2_clin_IMvigor210.

### Calculation of Phagocytosis Fraction and Its Correlation With Gene Expression (Hallmark)

To investigate the overall expression of PRFs in the samples, a GSVA (Gene Set Variation Analysis) enrichment analysis was performed using the R package. GSVA is a non-parametric and unsupervised method used to estimate activity changes in pathways and biological processes in samples. A total of 173 PRFs obtained from the literature were used to run the GSVA analysis and cor. test was used to calculate the correlation between each phagocytic factor and enrichment score (i.e., Pearson’s correlation coefficient).

### Analysis of Differentially Expressed Genes (Using The Cancer Genome Atlas Data)

We used limma in the R package to screen differentially expressed genes between normal and PRAD samples with the screening criteria of |logFC| >0.58 and FDR <0.05, and heat maps were constructed to show the overall expression of phagocytic factors in both groups of samples.

### Functional Enrichment Analysis

The Gene Ontology function and KEGG pathway enrichment analyses of the phagocytic factors were performed using clusterProfiler in the R package. Meanwhile, the GSEA enrichment analysis was also performed for the differential genes between the high/low score groups.

### Construction and Validation of Phagocytosis Factor Signatures

First, a univariate Cox regression analysis was used to screen PRAD prognosis-related phagocytosis factors [corrected *p* value by Benjamini and Hochberg (BH)]. Then, PRAD prognostic factors were further screened using a Lasso regression analysis (R package: glmnet), and risk scores were calculated based on the expression of each candidate factor and Lasso regression coefficients: Riskscore=∑coefi×Expri . The PRAD samples were divided into high and low risk groups based on the median score (PRAD dataset), a principal component analysis (PCA) was performed using the stats in the R package, and visualization of the groupings was performed using Rtsnet-SNE in the R package. The best cut-off points for the expression for the survival analysis of each candidate gene were performed by survminer in the R package (immune datasets KIRC and BLCA), and a receiver operating characteristic (ROC) curve analysis was performed using survROC in the R package to assess the predictive power of the signature.

### Assessment of the Proportion of Immune-Infiltrating Cells

CIBERSORT (https://cibersort.stanford.edu/) is a method for the characterization of cell composition on gene expression profiles from complex tissues. The leukocyte signature gene matrix LM22, consisting of 547 genes, was used to distinguish 22 immune cell types, which included myeloid subpopulations, natural killer (NK) cells, plasma cells, naive and memory B cells, and seven T cell types. We used the CIBERSORT in the R package combined with the LM22 feature matrix to estimate the proportion of the 22 human hematopoietic cell phenotypes in the PRAD samples from TCGA. The sum of the proportion of all the estimated immune cell types for each sample was equal to one. In addition, to better assess the proportion of the 28 immune cell types in different subpopulations (e.g., bracketed myeloid subpopulations, NK cells, plasma cells, naive and memory B cells, and multiple T-cell types), we also independently obtained the proportion of immune cell infiltrates from PRAD samples from TCGA using a ssGSEA (single-sample gene set enrichment) analysis in the R package GSVA. Finally, phagocytic factor expression levels were calculated using the cor. test and Spearman’s correlation, and the significance of immune cell abundance was obtained based on CIBEROST calculations.

### Immunotherapy Analysis

Risk scores were calculated using the PMID32472114 dataset (a total sample of 312 cases) to validate the utility of risk scores in the immunization dataset.

### Correlation Between the PRF Genes and Drug Sensitivity

To investigate the effect of the PRF genes on classical drug therapy of PRAD and their relationship, 8 classic drugs were selected from the Genomics of Drug Sensitivity in Cancer (GDSC) database (https://www.cancerrxgene.org/) for further analysis. The half-maximal inhibitory concentration (IC50) is an important indicator for evaluating the efficacy of a drug or the response of a sample to treatment. Using the largest publicly available pharmacogenomics database GDSC, the sample-based transcriptome predicts the response of each sample to the target and/or immunotherapy of PRAD. IC50 of the molecular-targeted drugs in the two groups was calculated through a ridge regression model constructed with the “pRRopheticl” package according to the cell expression profiles in the GDSC database. *p* < 0.05 indicates the statistical difference.

### Statistical Analysis

To determine the statistical significance of the risk score, the Wilcoxon test was used to compare differences between the two groups of samples. For plot presentation, where ns indicates *p > 0.05*, * indicates *p* < 0.05, ** indicates *p* < 0.01, *** indicates *p* < 0.001, and **** indicates *p* < 0.0001. Survival curves for the prognostic analysis were generated by the Kaplan–Meier method, and the significance of differences was determined using the log-rank test. Prediction of prognosis by the risk score was evaluated by ROC, and the area under the curve (AUC) was quantified using survival ROC in the R package.

## Results

### Phagocytosis Regulatory Factors Regulate Macrophage Phagocytosis and Participate in the Process of Tumor Development

**FIGURE 2:**
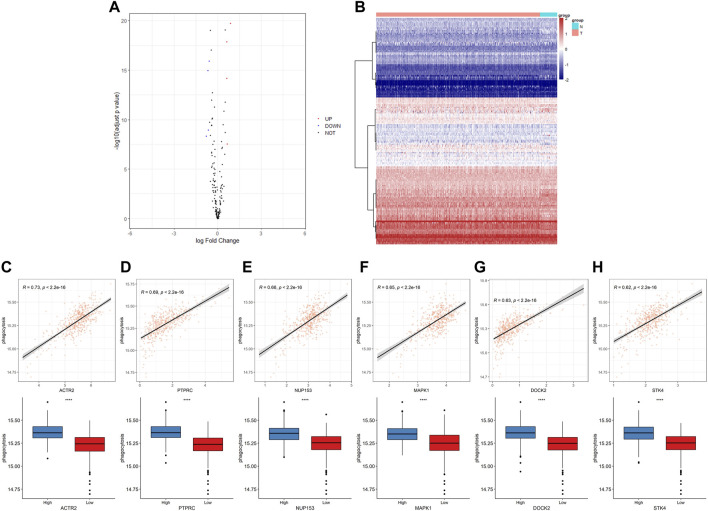
The expression of phagocytic factors and correlation with the phagocytic enrichment score in PRAD. **(A)** Analysis of phagocytic factor expression differences between normal and tumor samples (left: volcano diagram, red dots indicate up-regulation, blue dots indicate down-regulation); **(B)** Heat maps of the phagocytic factor expression in normal and tumor samples (right: red indicates the tumor group; blue indicates the normal group); **(C–H)** Correlation between 6 top phagocytic factors and phagocytic enrichment scores in PRAD samples (upper part); Comparison of the phagocytic enrichment score between the high- and low-expression group of top 6 top phagocytic genes (lower part).

First, we have summarized the enrichment of Phagocytosis Regulatory Factors (PRFs) in the TCGA–PRAD dataset (Supplementary Table S3) and their correlation with the expression of PRFs (Supplementary Table S4). The six genes with the highest correlation were significantly positively correlated with the enrichment scores, and the enrichment scores differed significantly among the groups with different gene expression levels (Figures 2C–H), implying that PRFs play an important role in PRAD.

To gain a deeper insight into the biological functions and pathways related to PRFs, GO and KEGG enrichment analyses were performed and the results showed that PRFs were mainly enriched to several biological processes including phagocytosis-related and energy metabolism processes, such as cellular phagocytosis and ATP synthesis (Supplementary Figures 1A–C; Supplementary Table S5_GO), and also involved in pathways such as oxidative phosphorylation (Supplementary Figure S1D; Supplementary Table S5_KEGG), which are all related to tumorigenesis development. Meanwhile, a GSEA enrichment analysis was also performed for the differential genes between high/low score groups. Our results showed that CELL-CYCLE and TGFB pathways were significantly enriched in the high-risk group (Supplementary Figure S2; Supplementary Table_deg.txt, Supplementary Table_deg_gsea.txt)

To further understand the role of PRFs in PRAD, we analyzed and compared the differences in expressions between 481 tumor samples and 51 normal samples in the TCGA–PRAD dataset (Supplementary Table S6) and found that only a few PRFs had significant differences (Figures 2A,B).

### Identification and Characterization of Prognosis-Related Phagocytosis Factors

**FIGURE 3:**
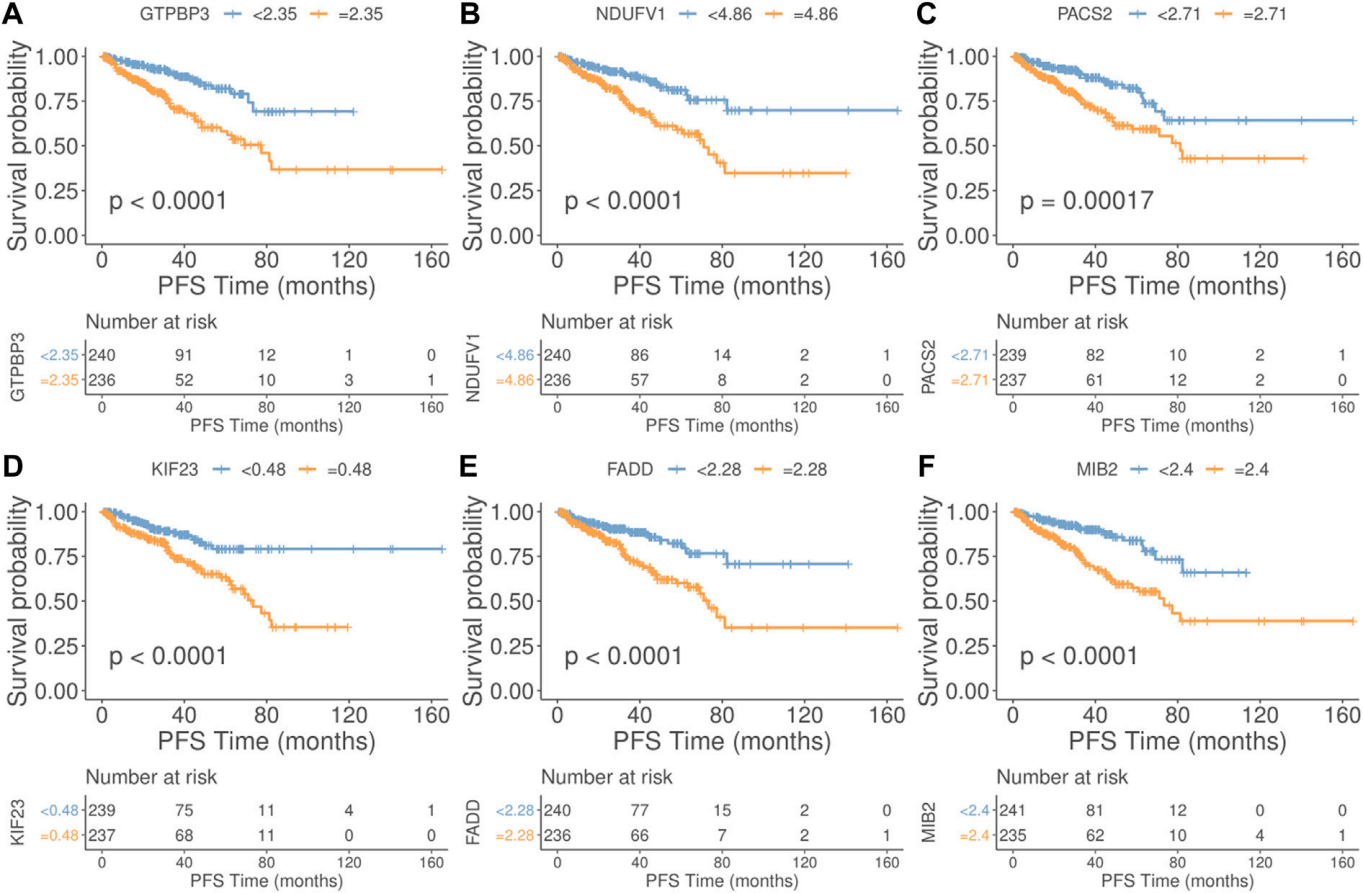
Relationship between the top 6 phagocytic factors and prognoses in PRAD. (Upper part of figure: Patient’s survival probability between different expression group at different PFS times; Vertical axis: Survival probability; Horizontal axis: PFS Time; Lower part of figure: Number of cases in different gene expression groups at different PFS Times. **(A)** GTBP3, **(B)** NDUFV1, **(C)** PACS2, **(D)** KIF23, **(E)** FADD, and **(F)** MIB2. (*p* < 0.0001, *p* < 0.0001, *p* = 0.00017, *p* < 0.0001, *p* < 0.0001, *p* < 0.0001, and *p* < 0.0001, respectively).

In our additional studies, we divided the samples into two groups according to the median expression of the PRFs, and the results from the survival analysis showed that 27 genes were significantly associated with overall survival (OS) in PRAD (Supplementary Figure S3, Supplementary Table S7). Also, the survival analysis of the six most relevant phagocytic factors (GTBP3, NDUFV1, PACS2, KIF23, FADD, and MIB2) in PRAD were conducted, and the results showed that the survival probability of the low expression group was significantly better than that of the high expression group at different PFS times (Figure 3) (*p* < 0.0001, *p* < 0.0001, *p =* 0.00017, *p* < 0.0001, *p* < 0.0001, *p* < 0.0001, and *p* < 0.0001, respectively).

### Signature Construction of the Phagocytosis Regulatory Factors

Using PRF genes which were significantly associated to prognosis in TCGA–PRAD as candidate feature genes, we finally constructed a prognosis signature containing 18 genes (Supplementary Figure S4; Supplementary Table S8) through the Cox–Lasso analysis. The samples were also divided into high-risk (238) and low-risk (238) groups according to their median values, and the information for high- and low-risk samples in score grouping is shown in Supplementary Table S9.

**FIGURE 4:**
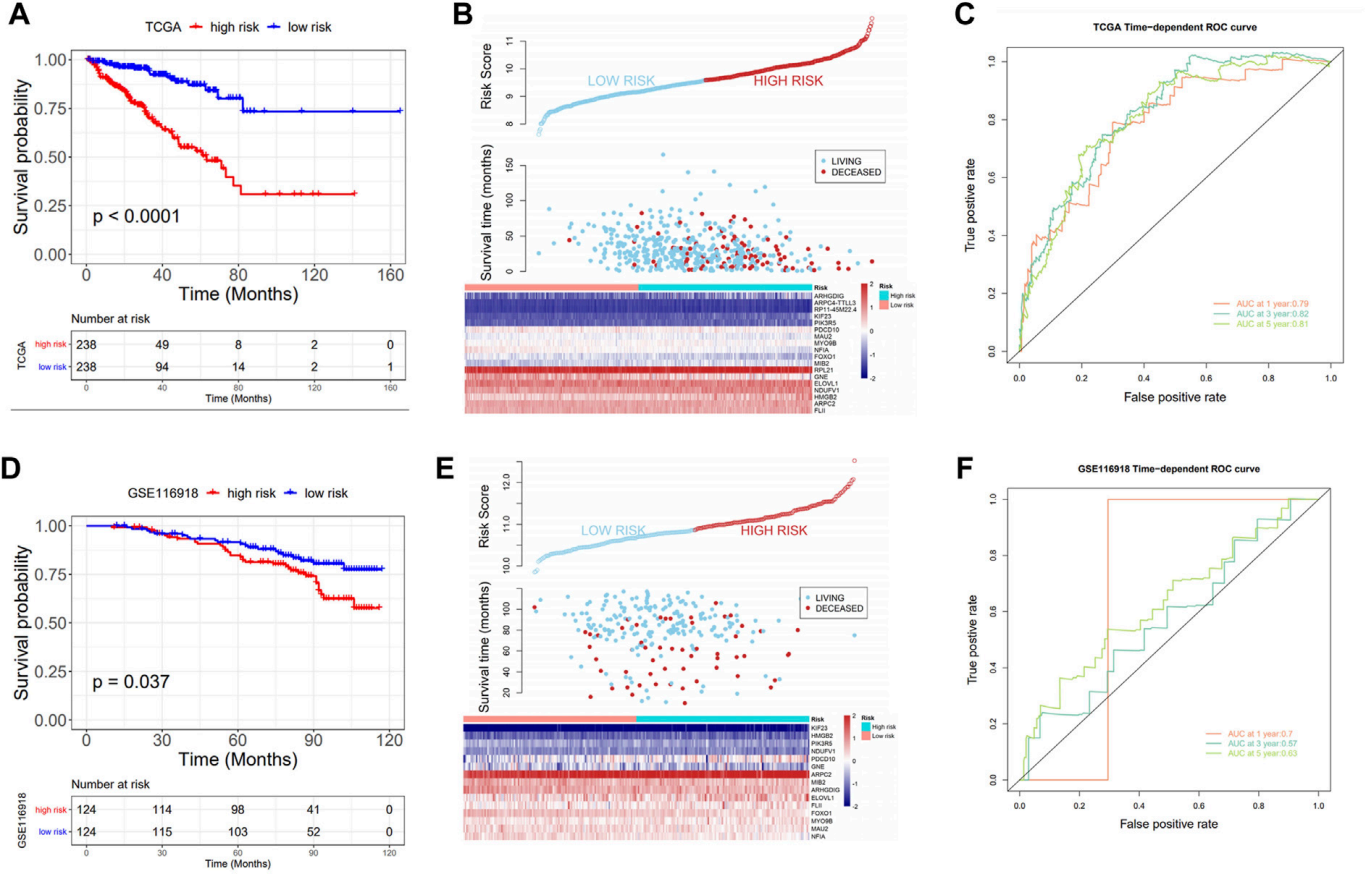
Prognostic value of the risk model. **(A)** KM curve: upper part: the probability of survival at different times in the high- and low-risk groups; lower part: the number of cases in the high- and low-score groups at different times; **(B)** Triple plot: upper part: risk scores for the high- and low-risk groups; middle part: survival time of the living and deceased cases; lower part: gene expression in the high- and low-score groups; **(C)** ROC analysis: assess the AUC values for OS predictive efficacy of risk scores; **(D–F)** Prognostic model validation in GSE116918: KM curve, triple plot, and ROC analysis.

Significant differences in prognoses were found between the high and low risk score groups using survival analysis, with the high-risk score group having a significantly inferior prognosis (Figures 4A,B) and ROC curves were used to assess the AUC values for OS predictive efficacy of the risk scores’ model (Figures 4A–C, 0.79 at 1 year, 0.82 at 3 years, and 0.81 at 5 years). Also, in the validation set GSE116918, the risk score was calculated using the risk score formula (Supplementary Table S10), and the AUC values for OS predictive efficacy of the risk scores’ model (Figure 4F, 0.7 at 1 year, 0.57 at 3 years, and 0.63 at 5 years)in the validation cohort, where the high-risk score group had a significantly inferior prognosis, and the prognostic efficacy was above 0.5 in all cases (Figures 4D–F).

### Signature of Phagocytosis Regulatory Factors Correlates With Patient Prognosis and Clinical Characteristics

**FIGURE 5:**
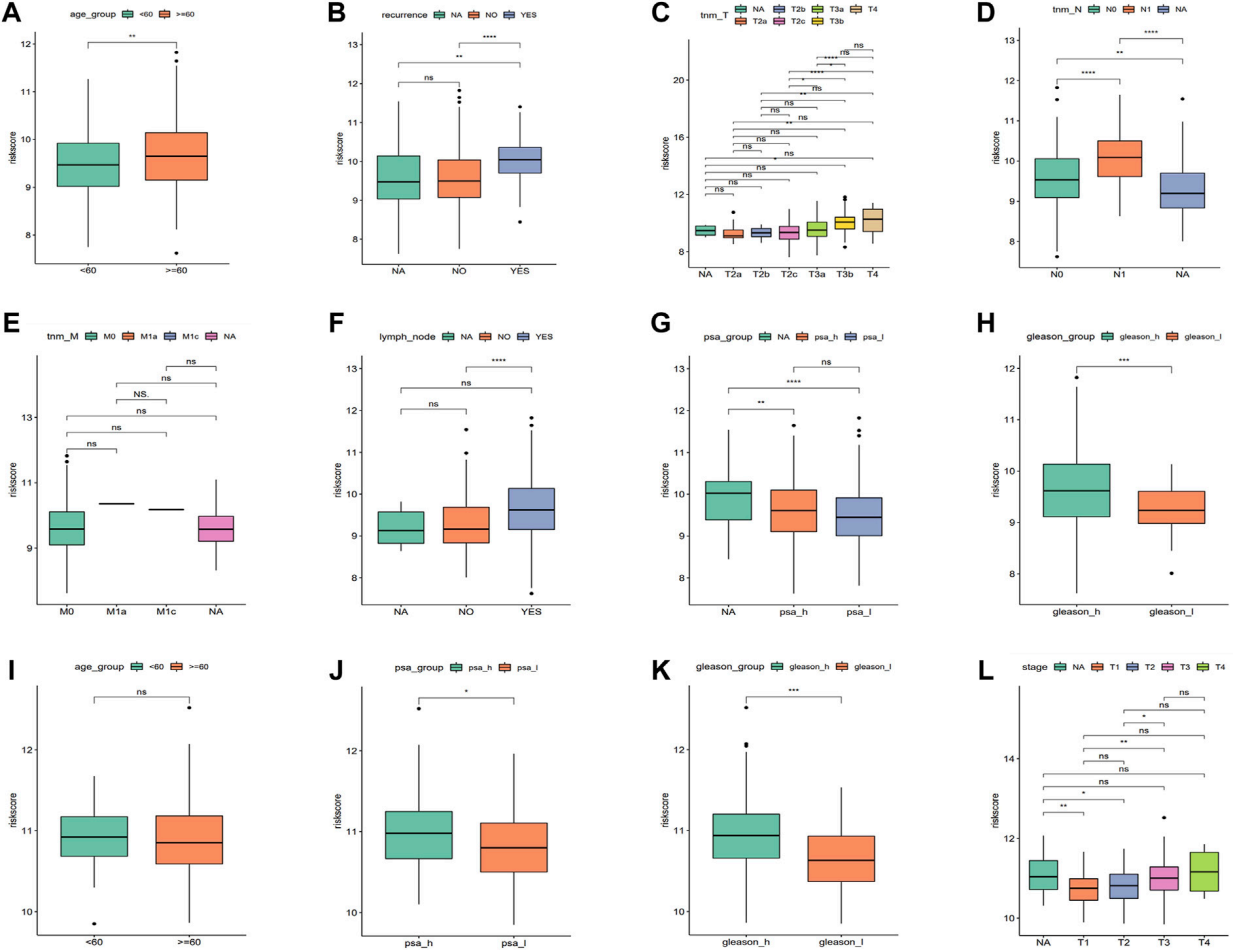
Correlation between the risk score with clinical characteristics. **(A–H)** Risk score comparison in different clinical characteristics in the training cohort (age, reccurrence, tnm_T, tnm_N, tnm_M, lymph_node, PSA, and Gleason, respectively. *p < 0.05, **p < 0.01, ***p < 0.001, ns: no significant). **(I–L)** Risk score comparison in different clinical characteristics in the validation cohort. (age, PSA, Gleason, and stage, respectively. *p < 0.05, **p < 0.01, ***p < 0.001, ns: no significant).

An analysis was conducted to determine the relationship between the risk score and the pathological features of PRAD, and we found a significant correlation as can be seen in Figure 5. The results showed significant differences for the risk score among clinical characteristic groups with PRAD such as age, recurrence, TNM stage, lymph node metastasis, PSA, and Gleason score in training cohort (Figures 5A–H). In addition, there were also significant differences in the risk score among the validation sets such as Stage, PSA, and Gleason scores in a validation cohort (Figures 5I–L), and the trends were consistent for both datasets. We found that the risk score for the stage 3/4 group was significantly higher than that in stage 1/2, and the same trend also existed for thevPSA and Gleason scores, that is, the risk scores of the high PSA group and the high Gleason score group were significantly higher than that of the low PSA group and the low Gleason group, respectively (Figure 5).

**FIGURE 6:**
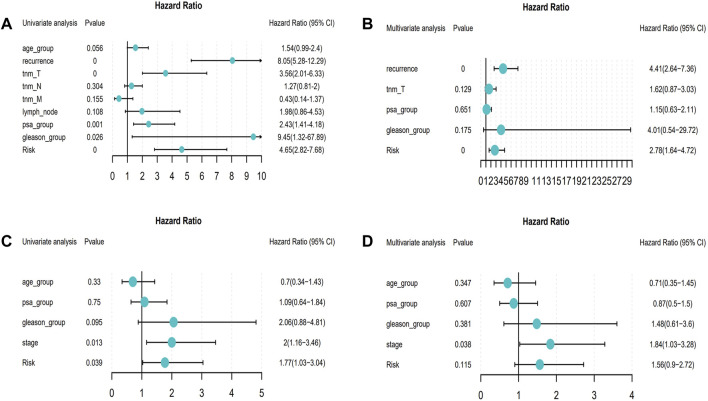
Forest map about the relationship between risk model and clinical characteristics (From left to right: Column 1: Clinical features; Column 2: *p* value; Column 3: Hazard Ratio forest map; Column 4: hazard ratio (HR) analysis with 95% confidence intervals (CI).). **(A)** Univariate Cox of model risk and eight clinical characteristics (age, recurrence, tnm_T, tnm_N, tnm_M, lymph_node, PSA, and Gleason) in the training cohort; **(B)** Multivariate Cox of recurrence, tnm_T, PSA, Gleason, and model risk in training cohort; **(C)** Univariate Cox of model risk and four clinical characteristics (Age, PSA, Gleason, and Stage) in the validation cohort; **(D)** Multivariate Cox of model risk and four clinical phenotypes (Age, PSA, Gleason, and Stage) in validation cohort.

**FIGURE 7:**
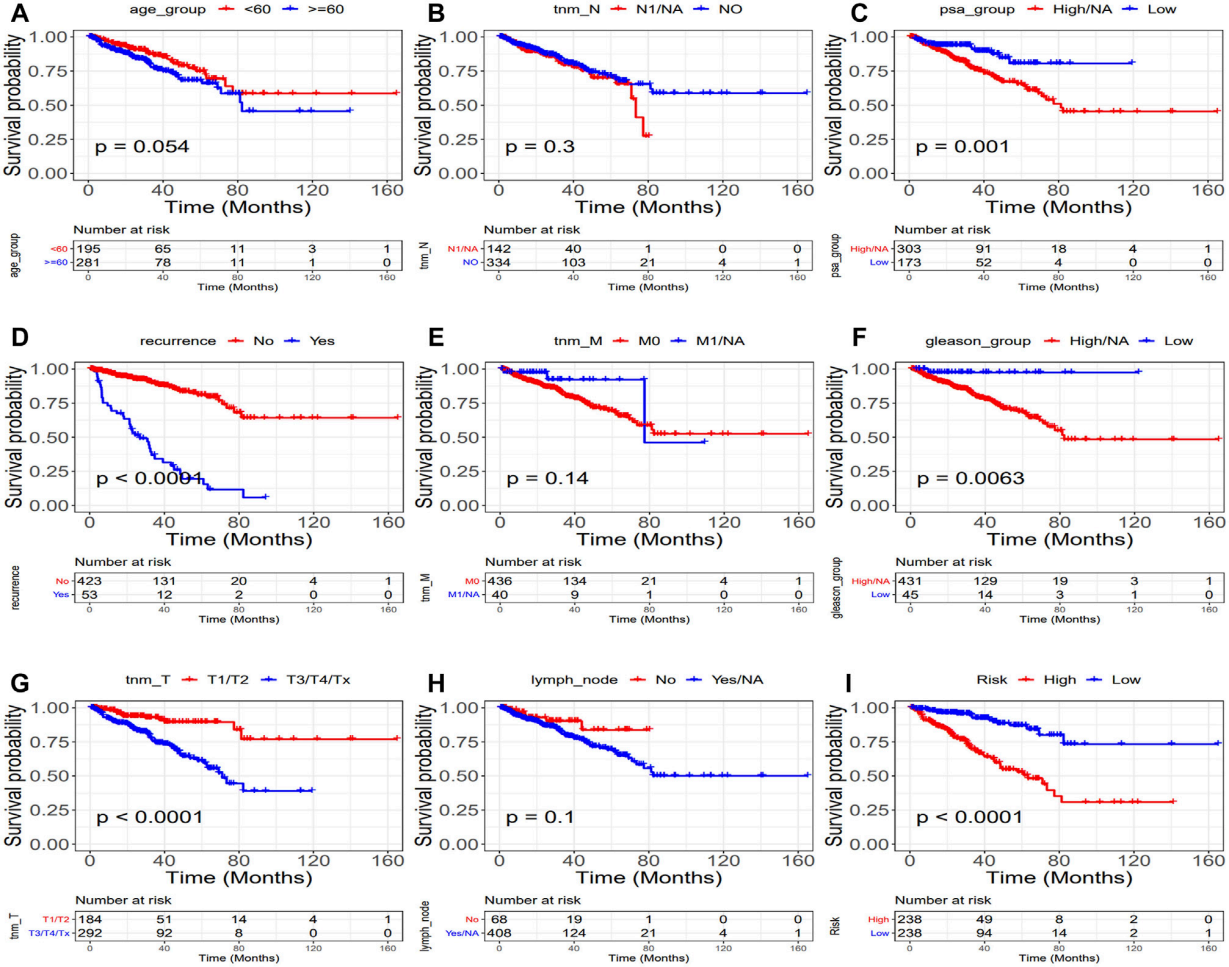
Relationship between clinical characteristics and prognosis (TCGA: PRAD). (Upper part of figure: Patient’s survival probability between different clinical features group at different times; Vertical axis: Survival probability; Horizontal axis: Time; lower part of figure: Number of cases in different groups at different Times.). **(A)** Age, **(B)** tnm-N, **(C)** PSA, **(D)** Recurrence, **(E)** tnm-M, **(F)** Gleason, **(G)** tnm-T **(H)** Lymph node, and **(I)** Risk. (*p* = 0.0054,*p* = 0.3, *p* = 0.001, *p* < 0.0001, *p* = 0.014, *p* = 0.0063, *p* < 0.0001, *p* = 0.1, and *p* < 0.0001, respectively).

The univariate and multivariate Cox regression analyses were performed to investigate the relationship between the risk score and clinicopathological features of the PRAD patients and results from the univariate analyses showed that the clinicopathological features (recurrence, TNM-T, PSA, and Gleason) and the risk score correlated with the survival of the patients (Figures 6A,C). Moreover, the results from the multivariate analyses demonstrated that the risk score was an independent risk factor correlated with the overall survival in both training cohorts (Figure 6B; Supplementary Tables S11, S12). After correcting other confounders, the risk score still proved to be an independent predictor of OS in the multivariate Cox regression analysis in the training set, and this trend was not consistent in the validation set (Figure 6D; Supplementary Tables S11, S12). Furthermore, we focused on the correlation between the clinical characteristics and the prognosis in the training set (TCGA–PRAD) (Figure 7).

### Relationship Between Classic Drugs and Phagocytosis Regulatory Factors

**FIGURE 8:**
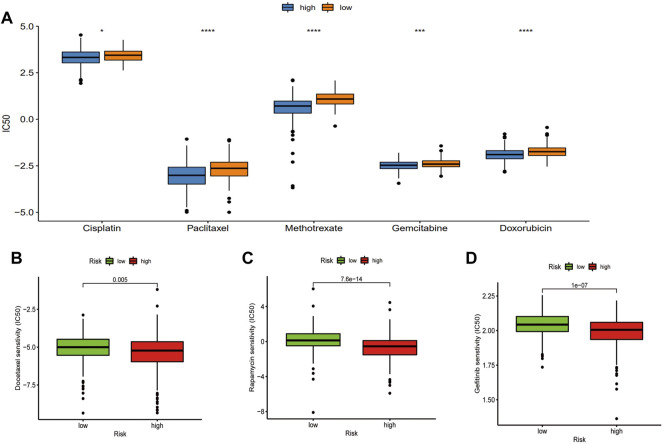
Relationship between classic drugs and PRFs. **(A)** The IC50 of Cisplatin, Paclitaxel, Methotrexate, Gemcitabine, and Doxorubicin in the high- and low-risk score groups. **(B)** The IC50 of Docetaxel in the high and low riskscore group. **(C)** The IC50 of Gefitinib in the high- and low-risk score groups. **(D)** The IC50 of Repamicin in the high- and low-risk score group (*p < 0.05, **p < 0.01, ***p < 0.001, ns: no significant).

To clarify the correlation between the PRFs and classic drugs for PC, Genomics of Drug Sensitivity in Cancer (GDSC) was used to investigate in-depth the drug sensitivity between the two risk score groups using the pRRophetic package in R software. A drug sensitivity analysis was performed to estimate the IC50 values of the drugs for each group. A lower IC50 always indicates better drug efficacy. We evaluated the relationship between PRFs riskScore and Cisplatin, Paclitaxel, Methotrexate, Gemcitabine, Doxorubicin, Docetaxel, Gefitinib, and Repamicin in PRAD, and the results showed lower IC50 values for these drugs in the high-risk group, suggesting a good result in the high-risk group with treatment of the aforementioned drugs (Figure 8, Supplementary Figure S5; Supplementary Table_IC50.txt). Based on the aforementioned results, we may have a better understanding of the effects of these drugs between the two groups, which will help to establish precise treatments for PRAD in the future. For example, if a patient with PRAD has a high PRFs riskScore, he can be combined with Docetaxel (or Cisplatin/Paclitaxel/Methotrexate/Gemcitabine/Doxorubicin) in addition to effective treatments such as surgery, endocrine therapy or radiation therapy, and may achieve a better prognosis.

### Signature of Phagocytosis Regulatory Factors Correlates With the Immune Microenvironment and Immunotherapy

**FIGURE 9:**
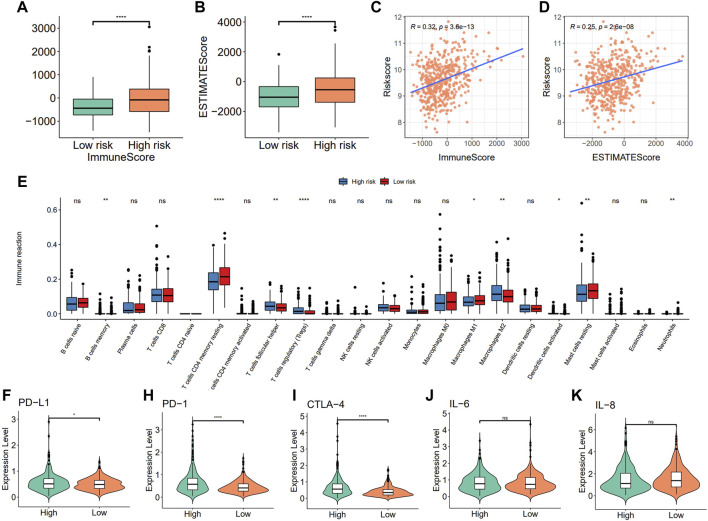
Correlation between the risk model with immune infiltration. **(A)** Differences in immune score; **(B)** estimated score; **(C,D)** Risk score is correlated with the immune score and estimated score; **(E)** immune infiltration between high- and low-risk groups; **(F–K)** Comparison of immune checkpoints (PD-L1, PD1, and CTL-4) and pro-inflammatory factor (IL-6 and IL-8) expression between high- and low-risk groups.

To further explore the relationship between the risk score and immune microenvironment, we first assessed the immune scores and estimated scores for the samples using ESTIMATE and the results showed that there were significant differences between the risk score groups (Figures 9A,B, *p <* 0.05, Wilcoxon test, Supplementary Table S13) and that they were both positively correlated with the risk score (Figures 9C,D). Furthermore, differences in the different categories of immune infiltration between high- and low-risk score groups were assessed and their results showed that there were significant differences in most categories of immune infiltration. It is interesting that there were significant differences between the high- and low-risk score groups about the macrophages M1 and M2 but with the opposite trend (Figure 9E, *p <* 0.05, Wilcoxon test).

To further characterize the correlations between the risk score and immunity, differential expression analysis of immune checkpoints and pro-inflammatory factors between high- and low-risk score groups were assessed using the Wilcoxon test, and results showed that the expression of the immune checkpoint genes PD-L1, PD-1, and CTLA-4 were significantly higher in the high-risk score group when compared to the low-risk score group, while there were no significant differences in the expression levels of pro-inflammatory factors such as IL-6 and IL-8 (Figures 9F–K).

**FIGURE 10:**
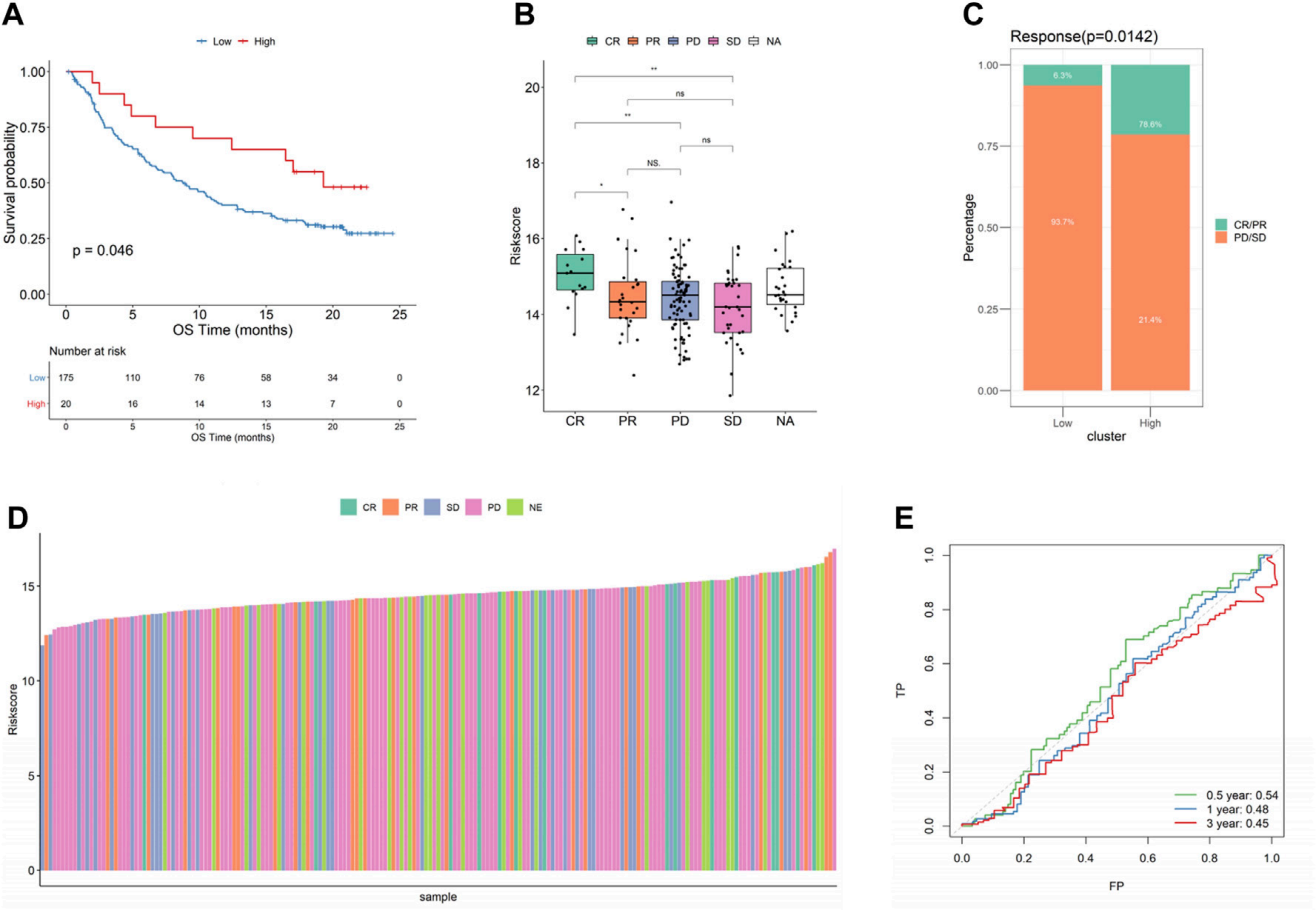
Relationship between risk score and effect of immunotherapy in bladder cancer. **(A)** KMcurve; **(B)** Riskscore distribution of different efficacy groups; **(C)** Distribution of different immune efficacy groups in the high- and low-risk score groups; **(D)** Risk score histogram of different efficacy groups; **(E)** ROC curve at different times (bladder cancer).

To investigate the effects of immunotherapy in high- and low-risk score groups, we calculated the risk score using the BLCA dataset (*n* = 195 cases) in IMvigor210CoreBiologies and the KIRC dataset (*n* = 311 cases) in PMID32472114, and divided the samples into high- and low-risk score groups and found that significant differences and consistent trends in the optimal threshold risk score groups and survival analyses in the prognosis between both high- and low-risk score groups, with the high-risk score group having a significantly inferior prognosis, which may reflect a better treatment effect of immunotherapy for patients in the high-risk group (Figure 10A, Supplementary Figure S5). After this, by comparing the risk scores of the different efficacy groups, we found that CR/PR was higher in bladder cancer than in the PD/SD group, and the composition of the immunotherapy efficacy categories also differed significantly within the high- and low-risk score groups (*p* < 0.05, Figures 10B–D), while there was almost no significant difference in renal cancer (Supplementary Figure S5B–D). Finally, a ROC curve analysis was conducted to investigate the prognostic prediction efficiency for bladder cancer at 0.5, 1, and 2 years with the new survival model, and the result showed that the model was better at indicating a prognosis within 6 months of OS for bladder cancer (Figure 10E). We also analyzed the predicted prognosis of kidney cancer at 1, 3, and 5 years, and the results showed that the areas under the ROC curves in the survival model were 0.58, 0.57, and 0.58, respectively (Supplementary Figure S5E).

### Model Verification Based on Relevant Immunohistochemical Results in the Human Protein Atlas Database

**FIGURE 11:**
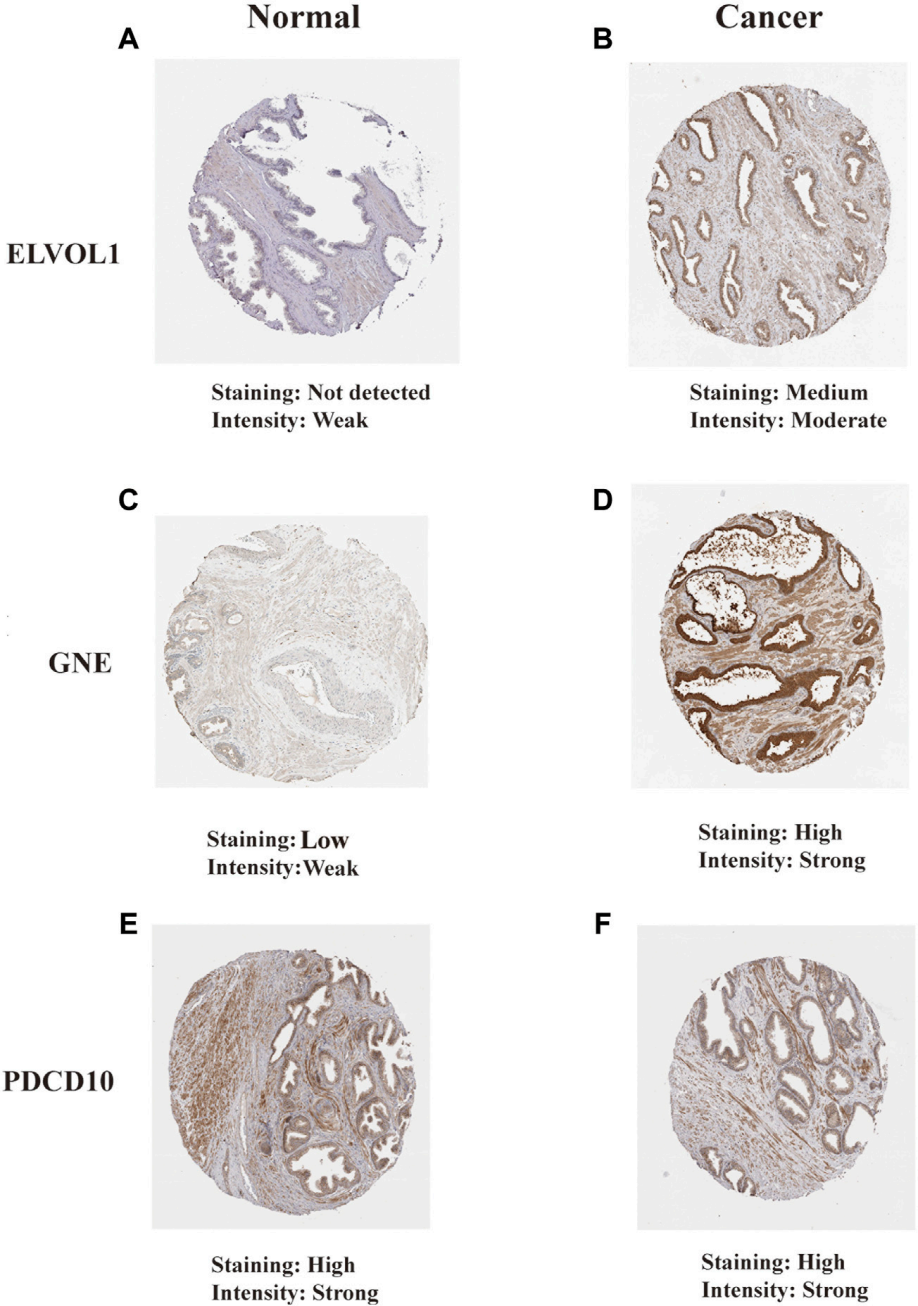
Translational levels of the signature genes in the Human Protein Atlas (HPA) database. **(A)** ELVOL1 in normal prostate tissue; **(B)** ELVOL1 in prostate cancer tissue; **(C)** GNE in normal prostate tissue; **(D)** GNE in prostate cancer tissue; **(E)** PDCD10 in normal prostate tissue; and **(F)** PDCD10 in prostate cancer tissue.

The Human Protein Atlas (HPA) (https://www.proteinatlas.org/) is a proteomics database that provides information on the organization and cell distribution of 26,000 human proteins. To analyze the translational levels of the PRF signature genes, the HPA database was used to determine the protein expression of 18 PRF characteristic genes. Our results showed that 2 of the 18 PRF characteristic genes (PDCD10 and GNE) were found to be over-expressed in prostate tissues, 7 moderately expressed, 3 poorly expressed, and the remaining 6 were not detected (Supplementary Table_coef_gene_HPA). Further studies showed that ELVOL1, PDCD10, and GNE proteins were also significantly over-expressed in prostate cancer (Figure 11). For GNE, prostate cancers and few cases of colorectal cancers displayed strong cytoplasmic staining. A majority of the cells showed weak to moderate granular cytoplasmic positivity. Squamous cell carcinomas, gliomas, renal, and gastric cancers were generally negative. For PDCD10, most malignant tissues showed a weak to moderate cytoplasmic immunoreactivity. Cases of prostate and liver cancers showed strong cytoplasmic staining (Supplementary Figure S6).

## Discussion

Currently, the consensus suggests that the incidence of PC is mainly related to the inactivation of tumor suppressor genes and the activation of oncogenes caused by persistent genetic and epigenetic variation. However, pathogen infection, physical and chemical factors, diet and many other factors related to exposure are also closely related to the incidence of PC (Johny et al., 2007; Kotb et al., 2015). Gollapudi et al. (2013) analyzed TAM density in benign prostatic hyperplasia, prostate intraepithelial neoplasia, and PC tissues, and found that their levels in PC were significantly higher than that in benign prostatic hyperplasia and prostate intraepithelial neoplasia. Moreover, the TAM density in Gleason Stage 4 patients was significantly higher than that in Gleason Stage 3 patients. In addition, Hu et al. (2015) found that the concentration of TAMs in PC tissues with distant metastasis was significantly higher than in those of patients with no distant metastasis. Gannon et al. (2009) found that the CD68 ^+^ macrophage number was significantly increased after ADT treatment using an optimized computer-aided statistical analysis. Therefore, the aforementioned study indicated that the increased density of TAMs in the prostate tissue may be involved in the development of PC and is closely related to castration resistance. Tumor-associated inflammation is one of the key features of cancer and phagocytic factors, chemokines and lymphokines are involved in the mediation and regulation of inflammation (Dvorak, 2015; Yasmin et al., 2015). There exists complex relationships among various cytokines as well as between their membrane receptors and soluble receptors, such as mutual coordination, inhibition, and antagonism, forming a cytokine regulatory network. The malignant tumor cytokine network is complex and plays an important role in various factors associated with tumor biology (Madalyn et al., 2017) and TAMs are a source of various cytokines. Like macrophages, cytokines such as PRFs may play an important role in the occurrence and development of tumors. However, to date, the mechanism action of PRFs in prostate cancer has not been reported. We are the first to demonstrate an enrichment of PRFs in PRAD and their correlation with the expression of PRFs. These results demonstrated that the genes with the highest correlation were significantly positively correlated with enrichment scores, implying that PRFs played an important role in PRAD. Furthermore, we went on to compare the differences in expressions between tumor samples and normal samples in PRAD and found that some phagocytic factors had higher expression levels in tumor samples when compared to normal samples. As a PRF, TIMMDC1 (also termed C3 or f1) was found to be significantly increased in metastatic lung cancer when compared to non-metastatic lung cancer (Wu et al., 2014). Liu et al. (2018) found that TIMMDC1 could regulate the Akt/GSK-3β/β-catenin signaling pathway in gastric adenocarcinoma SGC-7901 and BCC-823 cells, and downregulate the expression of TIMMDC1, thus reducing the mitochondrial energy metabolism and glycolysis of gastric cancer cells as well as inhibiting the proliferation, migration, and invasion of these cells (Liu, 2018). As a member of the ATPR (4-amino-2-trifluoromethylphenyl Retina) family of proteins, RPL21 is involved in the metabolism, as well as the PI3K-Akt and TGF-β signaling pathways and other cancer-related signaling pathways. It strongly inhibits proliferation and can induce the differentiation of tumor cells (Feng et al., 2019; Li et al., 2020). Consistent with previous studies, our results demonstrated that PRFs in PC were mainly enriched to several biological processes such as phagocytosis-related and energy metabolism, such as cellular phagocytosis and ATP synthesis, as well as pathways including oxidative phosphorylation, which are all related to the development of tumorigenesis in PRAD.

Cancer is known to have six characteristics: the ability to self-induce proliferation; insensitive to anti-proliferative signaling molecules; anti-apoptosis; unlimited proliferation potential; the ability to promote angiogenesis; and the ability to infiltrate and metastasize surrounding tissues. PRFs, as common cytokines, are expressed in almost all cancers and promote all six characteristics of cancer cells, thus promoting tumorigenesis. The tumor microenvironment provides the necessary immunosuppressive and hypermetabolic environment for tumor growth and progression, and TAMs play an important role in the inhibition of tumor immunity, while PRFs play important roles in the regulation of tumor immune cell induction, especially macrophages and T cells. In a study from Nature, researchers used intercellular CRISPR screening to identify PRFs (TIMMDC1, PDCD10, RPL21, GTPBP3, KIF23, PIK3R5, FOXO1, NFIA, ARPC2, HMGB2, NDUFV1, FADD, PACS2, HMHA1, CMC1, ANAPC7, and MIB2) in cancer cells. This work revealed an intrinsic cancer regulator that was sensitive to antibody-driven phagocytosis, expanding our understanding of the mechanisms by which cancer was resistant to phagocytosis by macrophages (Roarke et al., 2021). The programmed cell death molecule 10 (PDCD10), another PRF, is highly evolutionarily conserved and encodes for a gene that plays a role in the regulation of angiogenesis, proliferation, apoptosis, and migration of tumor cells (Lambertz et al., 2015). Over-expression of PDCD10 can increase proliferation and inhibit apoptosis of NSCLC A549 cells (Yang et al., 2018). Some studies have also found that PDCD10 expression was significantly increased in PC, and downregulation of PDCD10 expression could inhibit the proliferation and migration of these cells (Fu et al., 2016). The PRF GTPBP4 is a member of the GTPBP family, and its coding gene sequence is of highly conserved genes and widely distributed in various eukaryotes from yeast to humans (Young-Il et al., 2014). Recent studies have reported that the observed decrease in the expression of GTPBP4 could inhibit the proliferation of human colon cancer cells, while its increased expression was related to a decreased survival rate in colon and breast cancer patients (Andrea et al., 2010; Yu et al., 2016). The PRF FOXO1 (fork head frame transcription factor O1), also known as FKHR, plays an important role in cell proliferation and cancer biology (Kim et al., 2018) and its aberrant expression is associated with the occurrence of a variety of tumors (Shi et al., 2018); over-expression is associated with rhabdomyosarcoma (Ryder et al., 2017), breast cancer (Yu et al., 2019), and ovarian cancer (Han et al., 2019), and low expression is associated with colon cancer (Chae et al., 2019) and prostate cancer (Zhang et al., 2011). Studies have shown that the phagocytic regulatory factor NFIA was one of the key genes involved in the regulation of osteoclast formation and osteoclast activity, and the formation of bone metastasis of various tumors (Li et al., 2012). Actin-related protein complex2 (ARPC2) is one of the subunits associated with an actin-related protein2/3 (arp2/3) complex involved in the regulation of cytoskeletal motility and composition and participates in the regulation of cell growth, invasion, and other processes. ARPC2 expression is up-regulated in breast cancer, gastric cancer, and other tumor tissues, and the down-regulation of ARPC2 inhibits the invasion and migration of tumor cells, suggesting that it may be an oncogene (Zhang et al., 2017). In our study, the samples were divided into two groups (high- and low-risk score groups) according to the median expression of PRFs, and the results from the survival analysis showed that 27 genes were significantly associated with OS in PRAD. Furthermore, the survival analysis from the six most relevant phagocytic factors in PRAD were conducted and the results showed that six phagocytic factors were closely related to progression free survival (PFS) in PRAD patients, and PFS in the low-risk score group was significantly greater than that in the high-risk score group. Significant differences in prognoses were found between the high- and low-risk score groups based on the survival analysis, with the high-risk score group having a significantly worsening prognosis. Therefore, our results showed that there was a significant correlation between the risk score and clinical characteristics of PRAD such as age, recurrence, TNM stage, lymph node metastasis, PSA levels, and Gleason score. Moreover, the results from multivariate analyses demonstrated that the risk score was an independent risk factor correlated with the overall survival for PRAD in both the training datasets. Peng et al. (2014) reported a model based on the expression signature of VGLL3, IGFBP3 and F3. They found that compared with the prediction model that used only the clinical parameters, the addition of the tumor subtype classification could improve specificity of the overall survival of 5-year prediction. The area under the receiver operating characteristic curve value was increased from 0.755 to 0.815 in overall survival prediction, from 0.726 to 0.793 in PC-specific survival prediction, and from 0.730 to 0.793 in non-PC-specific survival prediction, respectively. Jhun et al. built a gene expression signature incorporated 49 genes including BUB1, CENPE, CENPF, DLGAP5, PRC1, and SMC4, which alone had an AUC of 0.68 and 0.76 for predicting recurrence and ML progression, respectively. Adding the signature to a logistic regression model that included clinicopathological factors could significantly improve the goodness of the fit of the model for recurrence and ML progression. The AUC increased by 3% and 6% for recurrence and ML progression, respectively (Jhun et al., 2017). In our study, the high risk score group having a significantly inferior prognosis and ROC curves were used to assess the AUC values for OS predictive efficacy of the risk scores’ model (0.79 at 1 year, 0.82 at 3 years, and 0.81 at 5 years, respectively.). However, in the validation cohort, the AUC values for OS predictive efficacy of the risk scores’ model was 0.7 at 1 year, 0.57 at 3 years, and 0.63 at 5 years, respectively. The ROC values about the PRF signature in the validation cohort set are not high enough. However, it is the first gene signature about PFs in PC, which can provide a new gene signature for predicting a prognosis of patients with PC. In the future, we could further explore how much predictive values the risk score would add to the model in addition to the clinical variables, such as comparing AUCs between the risk score and risk score + clinical variables.

Recent studies have shown that the occurrence and development of tumors are not only related to its genomic changes, but also affected by changes in TAM recruitment and polarization at the tumor immune microenvironment (Quail and Joyce, 2013; Hirsch et al., 2017; Wu and Dai, 2017; Alanazi et al., 2018). Therefore, understanding the tumor immune microenvironment may provide a new direction for the development of tumor therapy. TAMs with high density invasion in a variety of gynecological tumors, lung cancer, esophageal cancer, and other tumors are closely related to a poor prognosis (Krishnan et al., 2018; Li et al., 2018; Yagi et al., 2019). Therefore, TAMs may be a potential target for tumor therapy, and TAM-related therapeutic drugs have become an important area of research in recent years. TAMs are mostly derived from circulating monocytes, and recruitment of monocytes and macrophages is affected by signaling molecules secreted by tumor cells (Sica et al., 2008). Within the tumor microenvironment, vascular endothelial growth factor (VEGF), uPA, and other factors secreted by macrophages can promote tumor metastasis and new angiogenesis (Deng et al., 2018). Liu et al. (2018) demonstrated that apatinib, a small molecule inhibitor of vegFR-2, could significantly reduce the secretion of hepatocyte growth factor (HGF) from M2-type macrophages, thereby inhibiting Met phosphorylation in A549 and H1975 cells induced by M2-type macrophages. TAM-induced epithelial–mesenchymal transition (EMT) is attenuated by the hGF-MET signaling pathway. Conventional treatments for prostate cancer include radical prostatectomy, radiotherapy, chemotherapy, and endocrine therapy. Immunotherapy for prostate cancer is an emerging therapy, in which programmed cell death-1 (PD-1)/programmed cell death-ligand1 (PD-L1) inhibitors can improve the body’s anti-tumor immunity by activating its own immune system to cause tumor cell death, thus providing a new direction for immunotherapy in prostate cancer (Modena et al., 2016). Another immune checkpoint CTLA-4 inhibitor, ipilimumab has achieved good clinical efficacy in CRPC patients, with a PSA reduction of more than 50% in eight out of 50 patients (Slovin et al., 2013). Blocking the PD-1/PD-L1 immune checkpoint while blocking the CTLA-4 immune checkpoint may represent a novel, potential immunotherapy for prostate cancer. In our study, to further explore the relationship between the risk score and immune microenvironment, differences in different categories of immune infiltration between high- and low-risk score groups were assessed and results showed that there were significant differences in most categories of immune infiltration. Of note, it is interesting that there were significant differences between the high- and low-risk score groups about the macrophages M1 and M2 but with the opposite trend. The reason for this may be related to the different amounts of phagocytic factors released by the different types of macrophages, or the interference of phagocytic factors released by other cells. To further characterize the correlation between risk score and immunity, differential expression analysis of immune checkpoints and pro-inflammatory factors between high- and low-risk score groups was performed and the results showed that the expressions of the immune checkpoint genes PD-L1, PD-1, and CTLA-4 were significantly higher in the high-risk score group than in the low-risk score group, while there was no significant difference in the expression of pro-inflammatory factors such as IL-6 and IL-8. The aforementioned results suggested that immune checkpoint inhibitors or immune checkpoint inhibitors combined with endocrine therapy may achieve better results for patients with prostate cancer and a high-risk score, while for CRPC patients, CTLA-4 inhibitors or CTLA-4 inhibitors combined with endocrine therapy may prolong the survival of patients with a high-risk score.

Due to the heterogeneous nature of PC, it remains difficult for urologists to develop individualized clinical treatment plans for their patients (Knight, 2014). Although drugs such as docetaxel, abiraterone, and apatamide have been developed for the treatment of CRPC, which can extend life expectancy to some extent, the low survival rate of mCRPC suggests that it remains urgent for us to carry out further research into the correlation between the PRFs and classic drugs for PC. A lower IC50 always indicates better drug efficacy. In our study, we evaluated the relationship between PRF riskScore and Cisplatin, Paclitaxel, Methotrexate, Gemcitabine, Doxorubicin,Docetaxel, Gefitinib, and Repamicin in PRAD, and the results showed lower IC50 values for these drugs in the high-risk group, suggesting a good result in the high-risk group with treatment of the aforementioned drugs, which may make us have a better understanding of the effects of these drugs between the two groups. For example, if a patient with PRAD has a high PRFs riskScore, he can be combined with Docetaxel (or Cisplatin/Paclitaxel/Methotrexate/Gemcitabine/Doxorubicin) in addition to effective treatments such as surgery, endocrine therapy or radiation therapy, and may achieve a better prognosis.

To investigate the effect of immunotherapy on high- and low-risk score groups, we calculated the risk score through the BLCA dataset (*n* = 195 cases) and the KIRC dataset (*n* = 311 cases), and a survival analysis revealed significant differences in prognosis between both groups, with the high-risk score group having a significantly worse prognosis, which may imply a better treatment effect when immunotherapy is used for patients with a high-risk score. We also found that, by comparing the risk scores from different efficacy groups, we found that the CR/PR group was higher than the PD/SD group in bladder cancer, and the composition of immunotherapy efficacy categories also differed significantly between the high- and low-risk score groups, demonstrating that phagocytic factors were closely related to immunotherapy efficacy and highlighting their potential as therapeutic targets. Finally, the prognostic prediction efficiency for bladder cancer at 0.5, 1, and 2 years for the new survival model was better at indicating prognosis within 6 months of OS. We also analyzed the predicted prognosis for kidney cancer at 1, 3, and 5 years, and the result showed that the areas under the ROC curves for the survival model were 0.58, 0.57, and 0.58, respectively.

In conclusion, the multilayer changes in PRFs were related to prognoses of patients with PRAD and immune microenvironment and immunotherapy, which may provide us with a new directional approach for the future treatment of PRAD. An advantage of our study was that the data from multiple platforms were combined to construct a good indicator for use in guiding prognosis, drug treatment, and other aspects of the disease. However, it is worth noting that the immunization dataset was only clearly distinguishable with the best cut-off point, implying that only a small number of high-risk patients may benefit from immunotherapy.

## Data Availability

The data used to support the findings of this study are available from the corresponding authors upon request.

## Supplementary Material

Supplementary Figure 1 GO and KEGG enrichment analysis of phagocytic factor pathways. **(A)**. GO-BP; **(B)**. GO-CC; **(C)**. GO-MF and **(D)**. KEGG, respectively.

Supplementary Figure 2 GSEA enrichment analysis of phagocytic factor between high and low riskscore.

Supplementary Figure 3 Forest map of 27 prognostic phagocytic factors in PRAD.

Supplementary Figure 4 LASSO analysis. **(A)**. Forest map of OS model risk values and key genes; **(B)**. LASSO coefficient distribution of prognostic differential genes; **(C)**. Bar graph of correlation coefficients of 7 key genes.

Supplementary Figure 5 Relationship between risk score and effect of immunotherapy in Renal Cell Cancer. **(A)**. KM curve; **(B)**. Riskscore distribution of different efficacy groups; **(C)**. Distribution of different immune efficacy groups in the high and low Riskscore groups; **(D)**. Riskscore histogram of different efficacy groups; **(E)**. ROC curves at different times (Renal Cell Cancer).

Supplementary Figure 6 Translational levels of the signature genes in the Human Protein Atlas. **(A)**. GNE protein expression in cancers; **(B)**. PDCD10 protein expression in cancers.
