# Supplementary figures and images for: The Prognostic Signature and Therapeutic Value of Phagocytic Regulatory Factors in Prostate Adenocarcinoma (PRAD)
Source: Front Genet. 2022 May 30;13:877278. doi: 10.3389/fgene.2022.877278 (PMC9190300; doi:10.3389/fgene.2022.877278)

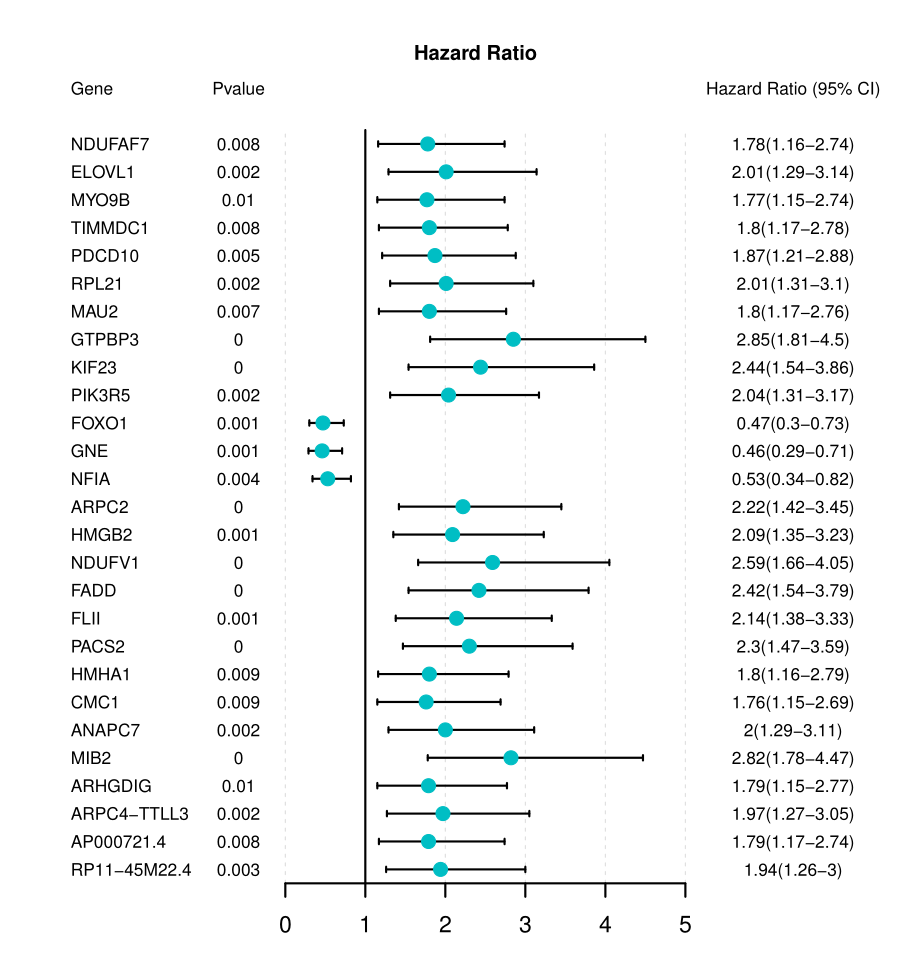

Supplement: Supplementary file 2 [file Image3.TIF]
